# Phellodendrine promotes autophagy by regulating the AMPK/mTOR pathway and treats ulcerative colitis

**DOI:** 10.1111/jcmm.16587

**Published:** 2021-05-18

**Authors:** Shuai Su, Xin Wang, Xiaonan Xi, Lanping Zhu, Qiuyu Chen, Hongxia Zhang, Yuan Qin, Boli Yang, Na Che, Hailong Cao, Weilong Zhong, Bangmao Wang

**Affiliations:** ^1^ Department of Gastroenterology and Hepatology Tianjin Medical University General Hospital Tianjin Institute of Digestive Disease Tianjin China; ^2^ State Key Laboratory of Medicinal Chemical Biology and College of Pharmacy Nankai University Tianjin China; ^3^ College of Life Sciences and Medicine Zhejiang Sci‐Tech University Hangzhou China; ^4^ Department of Pathology Tianjin Medical University Tianjin China; ^5^ Department of Pathology General Hospital of Tianjin Medical University Tianjin China

**Keywords:** AMPK/mTOR signalling pathway, autophagy, network pharmacology, phellodendrine, ulcerative colitis

## Abstract

To investigate the therapeutic effects of phellodendrine in ulcerative colitis (UC) through the AMPK/mTOR pathway. Volunteers were recruited to observe the therapeutic effects of Compound Cortex Phellodendri Liquid (Huangbai liniment). The main components of Compound Cortex Phellodendri Liquid were analysed via network pharmacology. The target of phellodendrine was further analysed. Caco‐2 cells were cultured, and H_2_O_2_ was used to stimulate in vitro cell model. Expression levels of LC3, AMPK, p‐AMPK, mTOR and p‐mTOR were detected via Western blotting and through immunofluorescence experiments. The therapeutic effects of phellodendrine were analysed via expression spectrum chip sequencing. The sequencing of intestinal flora further elucidated the therapeutic effects of phellodendrine. Compared with the control group, Compound Cortex Phellodendri Liquid could substantially improve the healing of intestinal mucosa. Network pharmacology analysis revealed that phellodendrine is the main component of Compound Cortex Phellodendri Liquid. Moreover, this alkaloid targets the AMPK signalling pathway. Results of animal experiments showed that phellodendrine could reduce the intestinal damage of UC compared with the model group. Findings of cell experiments indicated that phellodendrine treatment could activate the p‐AMPK /mTOR signalling pathway, as well as autophagy. Expression spectrum chip sequencing showed that treatment with phellodendrine could promote mucosal healing and reduce inflammatory responses. Results of intestinal flora detection demonstrated that treatment with phellodendrine could increase the abundance of flora and the content of beneficial bacteria. Phellodendrine may promote autophagy by regulating the AMPK‐mTOR signalling pathway, thereby reducing intestinal injury due to UC.

## 1 INTRODUCTION

Inflammatory bowel diseases, primarily Crohn's disease and ulcerative colitis (UC), are common gastrointestinal inflammatory diseases.^1^, ^2^ UC has become a global disease, the clinical interventions of which are mainly limited to anti‐inflammatory and immunomodulatory drugs.^3^ However, the progression and recurrence of UC cannot be completely controlled by existing therapeutic drugs, which also have many defects.^4^ Therefore, new prevention and treatment methods for the clinical treatment of UC must be developed.^5^

Cortex phellodendri has hypoglycaemic, antioxidant, anti‐inflammatory, hypotensive, immunomodulatory and other pharmacological activities.^6^, ^7^ Phellodendrine is one of the characteristic and most important active components of phellodendrone. Phellodendrine has the notable effects of lowering blood pressure, anti‐nephritis activity, inhibition of cellular immune responses and central inhibition.^8^, ^9^, ^10^ However, treatment of UC by using phellodendrine has not been reported yet.

Autophagy is a programmed cell degradation pathway mediated by lysosomes.^11^ Autophagy plays an important role in maintaining cell homeostasis, removing intracellular toxic substances and balancing metabolic energy.^12^ Autophagy is an important mechanism for the occurrence and development of UC,^13^ and it is closely related to disorders of inflammatory responses, invasion of pathogenic bacteria and repair disorders of intestinal mucosa.^14^, ^15^ Existing strategies for UC treatment can be optimized by further understanding the value of autophagy regulation in the prevention and treatment of this disease.^16^ Various traditional Chinese medicines with high efficacy against UC or their active components are reportedly involved in the regulation of autophagy.^17^ Such medicines and components provide new understanding and potential drugs for the development of clinical strategies for UC intervention.

As a key physiological energy sensor, adenosine monophosphate–activated protein kinase (AMPK) is a major regulator of energy balance in cells and organisms.^18^ AMPK co‐ordinates multiple metabolic pathways, balances energy supply and demand and ultimately regulates cell and organ growth.^19^ Mammalian target of rapamycin (MTOR) is a central regulator of cell growth and proliferation. MTOR forms two different complexes, namely mTORC1 and mTORC2.^20^ MTORC1 is regulated by various signals, such as growth factors, amino acids and cellular energy.^21^ Moreover, mTORC1 regulates numerous important cellular processes, including translation, transcription and autophagy. Regulation of energy metabolism balance is mediated by several related signalling pathways, wherein the AMPK/mTOR signalling pathways jointly constitute a switch of anabolic and catabolic processes in cells.^22^ MTOR is an important downstream signalling molecule of AMPK and plays a negative regulatory role in autophagy regulation.^23^, ^24^

From the perspective of network pharmacology, we explored the potential mechanism by which phellodendrine intervenes in regulating autophagy and preventing and treating UC via the AMPK‐mTOR signalling pathway. This study provides a theoretical basis for its clinical application.

## 2 METHODS

### 2.1 Clinical samples and patient information

A total of 40 patients with moderate left semicolon UC who were diagnosed and treated in the General Hospital of Tianjin Medical University from December 2018 to August 2020 were selected as the study patients. According to the random number table method, they were divided into mesalazine + normal saline enema group and mesalazine + Compound Cortex Phellodendri Liquid (Compound Cortex Phellodendri Liquid was kindly provided by Shandong Hanfang Pharmaceutical Co., Ltd [Chinese medicine character: Z10950097]). group (n = 20 cases in each group). In the mesalazine + saline enema group, 11 were males and 9 were females. The mean age was 39.62 ± 8.58 years. The mean course of disease was 5.31 ± 2.76 years. In the mesalazine + saline enema group, 10 were males and 10 were females. The mean age was 40.12 ± 9.06 years. The average course of disease was 5.18 ± 2.14 years. No statistically significant difference (*P* > 0.05) was observed between the two groups in terms of gender composition, average age, average course of disease and other general data, indicating comparability. The inclusion criteria were as follows: patients conformed to the consensus opinion on diagnosis and treatment of inflammatory bowel disease formulated by the Inflammatory Bowel Disease Group of The Chinese Medical Association in Beijing in 2018. Lesions were located in the left half of the colon, and the disease was mild to moderate. The patients had no obvious abnormality in the electrocardiogram, liver and kidney functions. The study was reviewed and approved by the medical ethics committee of the hospital, and the patients were made aware of the objectives and scope of the study and signed informed consent. The exclusion criteria were as follows: (a) patients with severe cardiovascular and cerebrovascular diseases, haematopoietic system, liver, kidney and other primary diseases; (b) severe complications, such as colonic haemorrhage, intestinal obstruction, intestinal perforation and toxic megacolon; (c) patients with other infectious or non‐infectious colitis; (d) women who are preparing for pregnancy or pregnant and breastfeeding; (e) allergic to Compound Cortex Phellodendri Liquid and amino salicylic acid drugs; and those who could not take medications as prescribed and thus efficacy could not be evaluated. For the evaluation of disease, clinical activity was graded using the Mayo score (MS) in patients with UC at baseline and at the end of the study at 12 weeks. Mayo score includes Mayo stool frequency subscore, Mayo rectal bleeding subscore, Mayo endoscopic subscore (MES) and Mayo Physician's global assessment subscore, and each subscore ranges from 0 to 3.^25^ According to *Chinese consensus on diagnosis and treatment of inflammatory bowel disease (Beijing, 2018)*, clinical response is classed into (a) ease: The clinical symptoms disappeared, and MES ≤ 1 point; (b) effective: The clinical symptoms basically disappeared, and MES ≤ 1 point; and (c) no improvement in clinical symptoms, and MES ≤ 3 point. Total effective rate means that the per cent of patients whose clinical response with ease and effective. Besides, samples for C‐reactive protein (CRP) and faecal calprotectin were collected to assess the inflammatory activity at baseline and at the end of the study at 12 weeks.

### 2.2 Animal models

In this study, SPF grade female C57BL/6 mice (18‐22 g) were selected as the animal model. According to weight, the mice were randomly divided into four groups with five mice in each group: normal control group, model group, positive control group and phellodendrine group. Phellodendrine was given at a dosage of 30 mg/kg. The positive control group was treated with salazosulphapyridine (40 mg/mL) at a dose of 200 mg/kg. The normal control and the model control groups were treated with double steam water. The acute phase model of UC was induced by 5% dextran sulphate sodium (DSS). During the adaptation period, the mice were freely given food and water. The experiment was began by replacing the drinking water with a 5% DSS solution (except for the normal control group) and giving the mice a free drink. The daily water intake of each mouse was calculated as 6 mL, and a sufficient amount of the DSS solution was added the next day. The DSS solution was administered for 8 days. After the initial administration of DSS for 24 h, soft stool, diarrhoea or blood stool was found in the mice, that is the mice were given the drug by gavage. The mice in the normal control and the model control groups were given 0.1 mL of double‐steamed water every day. The mice in the positive control group were given 0.1 mL salazosulphapyridine (40 mg/mL) every day. The phellodendrine group was administered with 30 mg/kg of phellodendrine. The overall performance of each mouse was assessed by disease activity index (DAI), including bodyweight loss, stool consistency and faecal blood, and the range of every item is from 0 to 4. The sum of all scores from these three parameters was calculated as the DAI.^26^ After 7 days of administration, all the mice were killed. The abdominal cavity of the mice was exposed, and their colon was dissected and its length was measured. The colon was cut lengthwise along the mesentery, and the stool was cleaned with normal saline and fixed with 10% neutral formalin solution. Gross injuries were observed and scored under a stereomicroscope, and severe ulcers were taken for pathological examination.

### 2.3 Histological detection of colon

Changes in colonic tissue mucosa and serosal surface were visually observed, and gross morphological changes were scored. A piece of fresh colon tissue was collected, and the size after dressing was about 1.0 cm × 1.0 cm × 0.2 cm. The samples were placed in an embedding box and fixed in 4% paraformaldehyde for 24 h. The samples (3‐4 μm thick) were embedded in paraffin and serially sectioned. Histomorphological changes were observed via haematoxylin‐eosin staining under a light microscope. Pathological changes in the tissues were observed and scored under a microscope.

### 2.4 Cell culture

Caco‐2 cells were routinely cultured in complete medium (DMEM containing 10% foetal bovine serum and 1% streptomycin‐penicillin solution) in an incubator at 37℃ and 5% CO_2_. The liquid was changed every other day and passed once every 3‐4 days.

### 2.5 Network pharmacology analysis

Active phellodendrine compounds were predicted via database collection, literature search and target prediction on the basis of ligand structural characteristics. Targets of active compounds were obtained from the BATMAN website (http://bionet.ncpsb.org/batman‐tcm/) gene target information. The possible targets were collected according to chemical structure similarity prediction. All target information was standardized using UniProt (http://www.uniprot.org/).

### 2.6 Gene Ontology analysis

Gene Ontology (GO) is the international standard classification of gene function. The selected genes were classified via GO analysis. Important biological functions were enriched via significance analysis, misjudgement rate analysis and enrichment analysis of discrete distribution. Using the GO online analysis tool DAVID (https://david.ncifcrf.gov/) (*P* < 0.5, Benjamini < 0.5), GO analysis was performed.

### 2.7 Pathway analysis

Pathway analysis was based on the selected genes according to the public database KEGG (https://www.genome.jp/kegg/). The pathway classification significantly related to the experimental purpose was obtained via significance analysis of discrete distribution. Omicshare (http://www.omicshare.com), an online pathway analysis platform, was used to obtain the pathway enriched information of phellodendrine therapy UC‐related genes with *P* < 0.5 and FDR < 0.05 as parameters.

### 2.8 Protein‐protein interaction (PPI) network construction

The interaction gene/protein retrieval tool STRING (https://string‐db.org/) was used to retrieve and predict gene/protein interactions on the basis of biological databases and Web resources. PPI network is an important tool for systematic understanding of cellular processes. This network can be used to filter and evaluate functional genomic data and provide a visual platform for annotating protein structures and functions. The PPI network for phellodendrine therapy UC‐related targets was explored to provide new directions for searching for significant efficacy targets and for future experimental validation of phellodendrine therapy UC‐related targets. Phellodendrine therapy UC‐related target PPI network data (SCORE ≥ 0.9) were obtained using the online database STRING, and cytoscape‐V3.6.1 was used to construct the visual PPI network diagram.

### 2.9 Western blot

Total intracellular proteins were extracted. Protein concentration was determined via the BCA method. First, 40 μg of the samples were isolated by 10% SDS‐PAGE. The isolated proteins were transferred onto a PVDF membrane. Then, 5% skimmed milk was sealed for 1 h. Primary antibody was added and incubated at 4℃ overnight. TBST was thoroughly washed three times for 10 min each time. The PVDF membrane was sealed into the secondary diluent. The membrane was incubated in a shaker at room temperature for 1 h and then washed with TBST three times for 10 min each time. A luminescent solution was prepared and evenly dripped on the PVDF membrane. GAPDH was used as an internal reference and repeated for three times. ImageJ software was used for grey value analysis.

### 2.10 Immunofluorescence

Cell slides were taken, sealed with 10% BSA, covered with primary antibody (1:1000) and incubated at 4℃ for 8 h. The slides were then placed in PBS (pH 7.4). The slides were shaken and washed in a decolourizing shaker three times for 5 min each time. After the cells slightly dried, a secondary antibody (1:500) was dropped to cover the cells. The cells were incubated at room temperature for 50 min. The cells were washed with PBS three times, and each slice was dripped with 50 μL of DAPI dye. The cells were then incubated at room temperature for 5 min. Finally, the samples were washed with PBS and observed under a fluorescence microscope. After images were taken, an image analysis software was used to analyse optical density value.

### 2.11 Expression spectrum chip sequencing

Intestinal tissues were collected from the animals. Total RNA was extracted via the TRIzol method. The total RNA was purified using RNeasy Mini Kit (Qiagen). After the total RNA was purified, the first and second strands of cDNA were synthesized in one step. A fluorescent dye (Cy3) was used to label and fragment cRNA. Finally, the cRNA was hybridized with a chip. The operation was conducted in accordance with Agilent chip instructions.

### 2.12 Analysis of intestinal flora

After the treatment, fresh faeces were collected from the corresponding colon of mice in each group and placed in a sterile frozen storage tube. The faecal samples were stored in a refrigerator at −80℃. The QIAamp faecal DNA extraction box was used to extract DNA. Absorbance (*A*) was determined using an ultraviolet spectrophotometer at 260 nm, and its concentration was calculated. Purity was determined on the basis of A_260_/A_280_ values. Integrity was verified by 0.8% agarose electrophoresis. Genomic DNA was amplified via PCR, and the primers used were specific primers of the bacterial 16S rDNA V4 region. A sequencing library was then constructed. High‐quality sequencing data were then analysed via bioinformatics methods.

### 2.13 Statistical analysis

SPSS 17.0 statistical software was used for data analysis. Measurement data are expressed as mean ± standard deviation. One‐way ANOVA was used for comparison between groups. Student's *t* test was used for comparison between two groups. *P* <.05 was considered statistically significant.

## 3 RESULTS

### 3.1 Retention enema of Compound Cortex Phellodendri Liquid combined with oral administration of mesalazine can significantly improve the clinical effects on patients with mild and moderate left semicolonic UC

**FIGURE 1:**
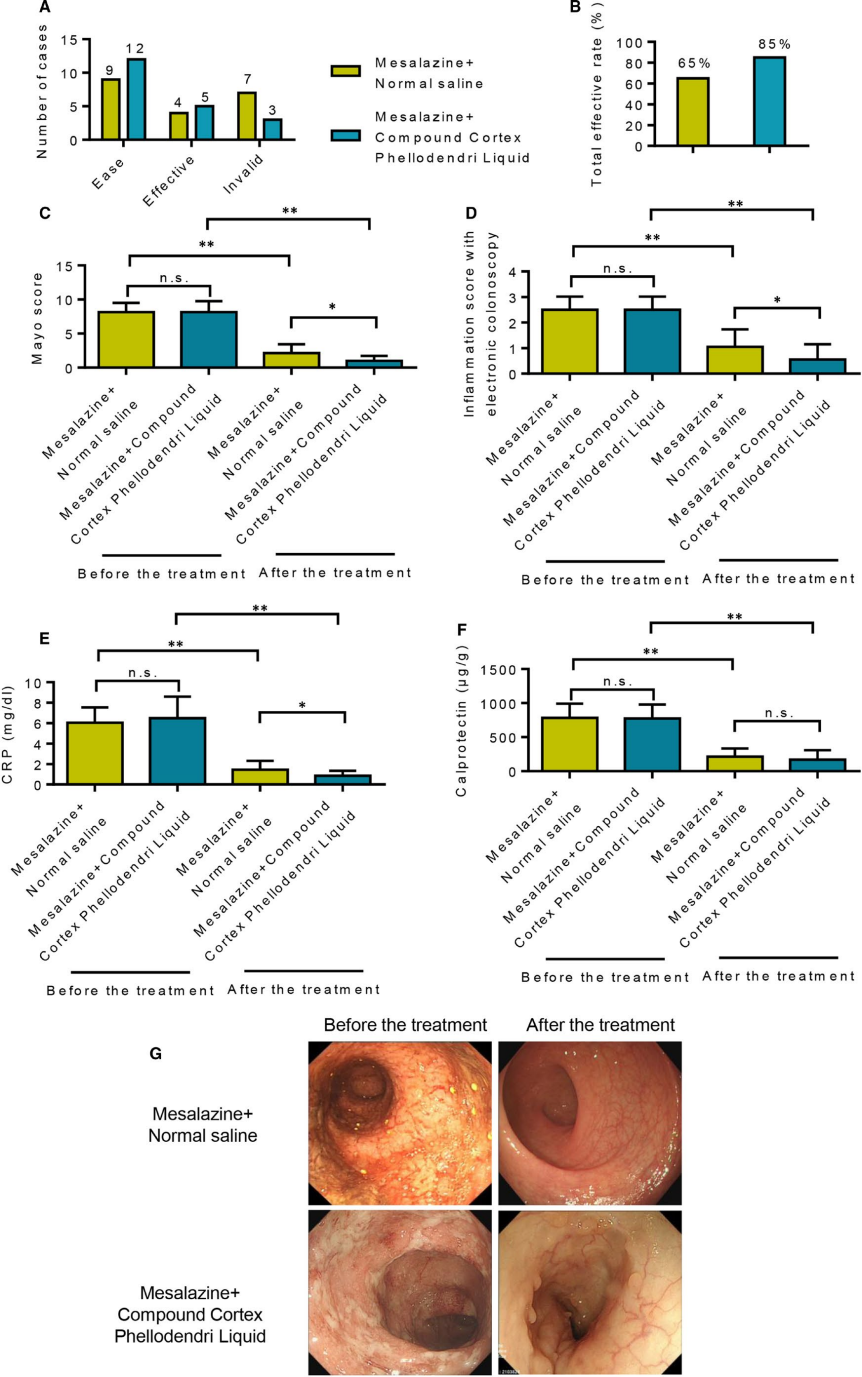
Clinical study of Compound Cortex Phellodendri Liquid combined with oral mesalazine for the treatment of ulcerative colitis. A, Patients in the mesalazine+saline enema group and the mesalazine + Compound Cortex Phellodendri Liquid group were relieved after 12 weeks of treatment. B, The total effective rate of the treatment for the patients in the mesalazine + saline enema group and the mesalazine + Compound Cortex Phellodendri Liquid group. C, Comparison of modified Mayo score before and after treatment between the two groups of patients. D, Results of electronic colonoscopy inflammation scores before and after treatment in the two groups. E, Comparison of CRP values between the two groups of patients before and after the treatment. F, Comparison of calprotectin levels before and after treatment in the two groups of patients. G, Colon improvement in the two groups of patients before and after the treatment. Data are presented as the means ± SD. *P < 0.05 and **P < 0.01

The therapeutic effects of Compound Cortex Phellodendri Liquid on ulcerative colitis were evaluated by composing groups administered with mesalazine alone and mesalazine combined with Compound Cortex Phellodendri Liquid. Results showed that the total effective rate of patients in the combined treatment group was higher than that in the mesalazine group after 12 weeks of comparative treatment, with statistically significant differences (*P* < 0.05) (Figure 1A and B ). By contrast, no statistically significant difference was found between the two groups in the modified Mayo score before treatment (*P* > 0.05). The modified Mayo score of the two groups after treatment was lower than that before treatment, and the difference was statistically significant (*P* < 0.05). However, the Mayo score in the mesalazine combined with Compound Cortex Phellodendri Liquid group was lower, indicating a better therapeutic effect (Figure 1C). Electronic colonoscopy inflammation analysis showed that the score of colon inflammation in both groups was lower after treatment than that before treatment, and the differences were statistically significant (all *P* < 0.05). In addition, compared with mesalazine alone, the inflammation score was lower in the combined mesalazine and Compound Cortex Phellodendri Liquid treatment (Figure 1D). A comparison of hs‐CRP and calprotectin levels between the two groups before and after treatment showed that the levels of both CRP and calprotectin significantly decreased after the treatment. However, the treatment group with mesalazine combined with Compound Cortex Phellodendri Liquid achieved better results (Figure 1E and 1F). The treatment effects of the two groups are shown in Figure 1G.

### 3.2 Network pharmacology analysis of the main components of Compound Cortex Phellodendri Liquid

**FIGURE 2:**
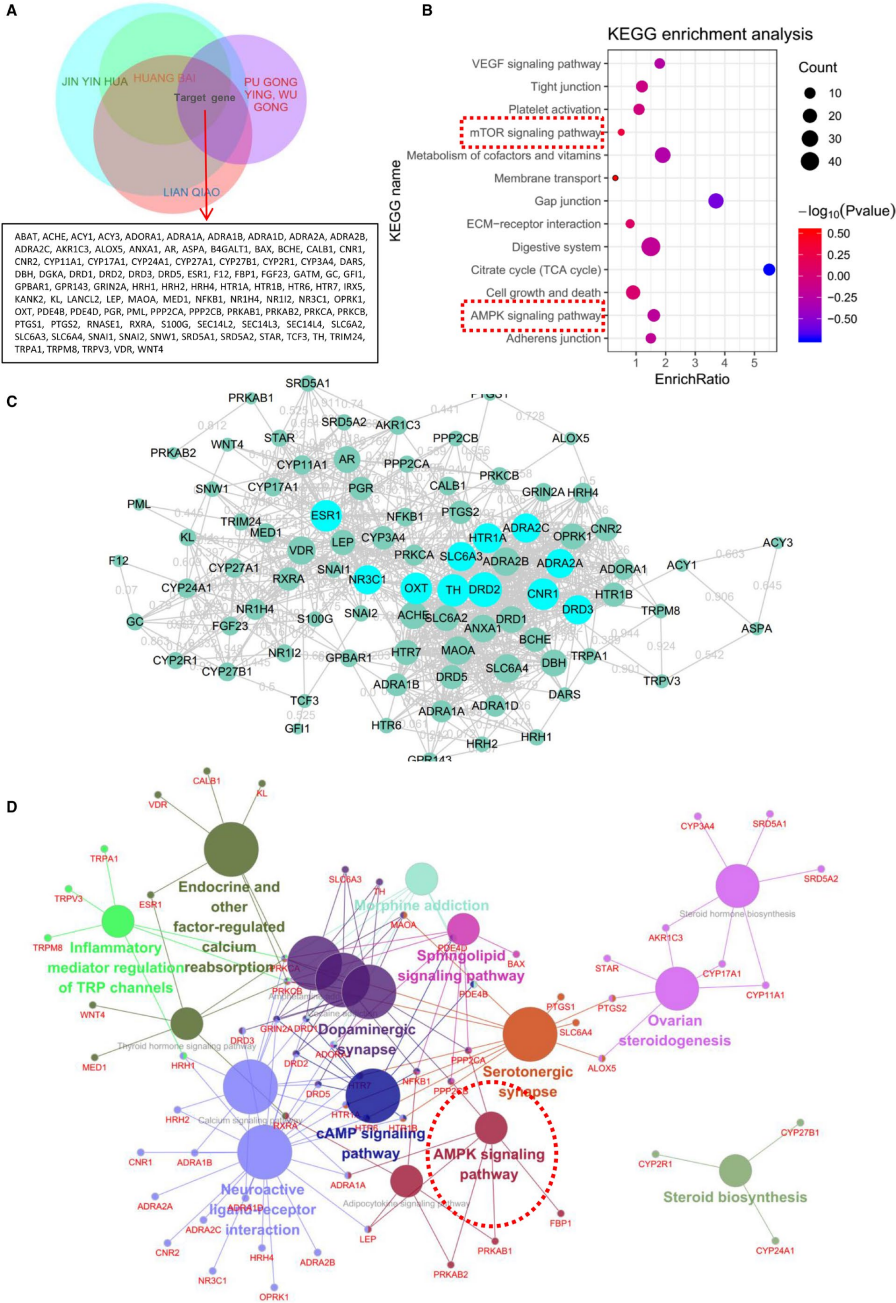
Network pharmacological analysis of the main components of Compound Cortex Phellodendri Liquid. A, Target prediction analysis of the main components of Compound Cortex Phellodendri Liquid. B, KEGG enrichment analysis of potential targets of the main components of Compound Cortex Phellodendri Liquid. C, Protein‐protein interaction network analysis of the potential targets of the main components of Compound Cortex Phellodendri Liquid. D, GO analysis of potential targets of the main components of Compound Cortex Phellodendri Liquid

**FIGURE 3:**
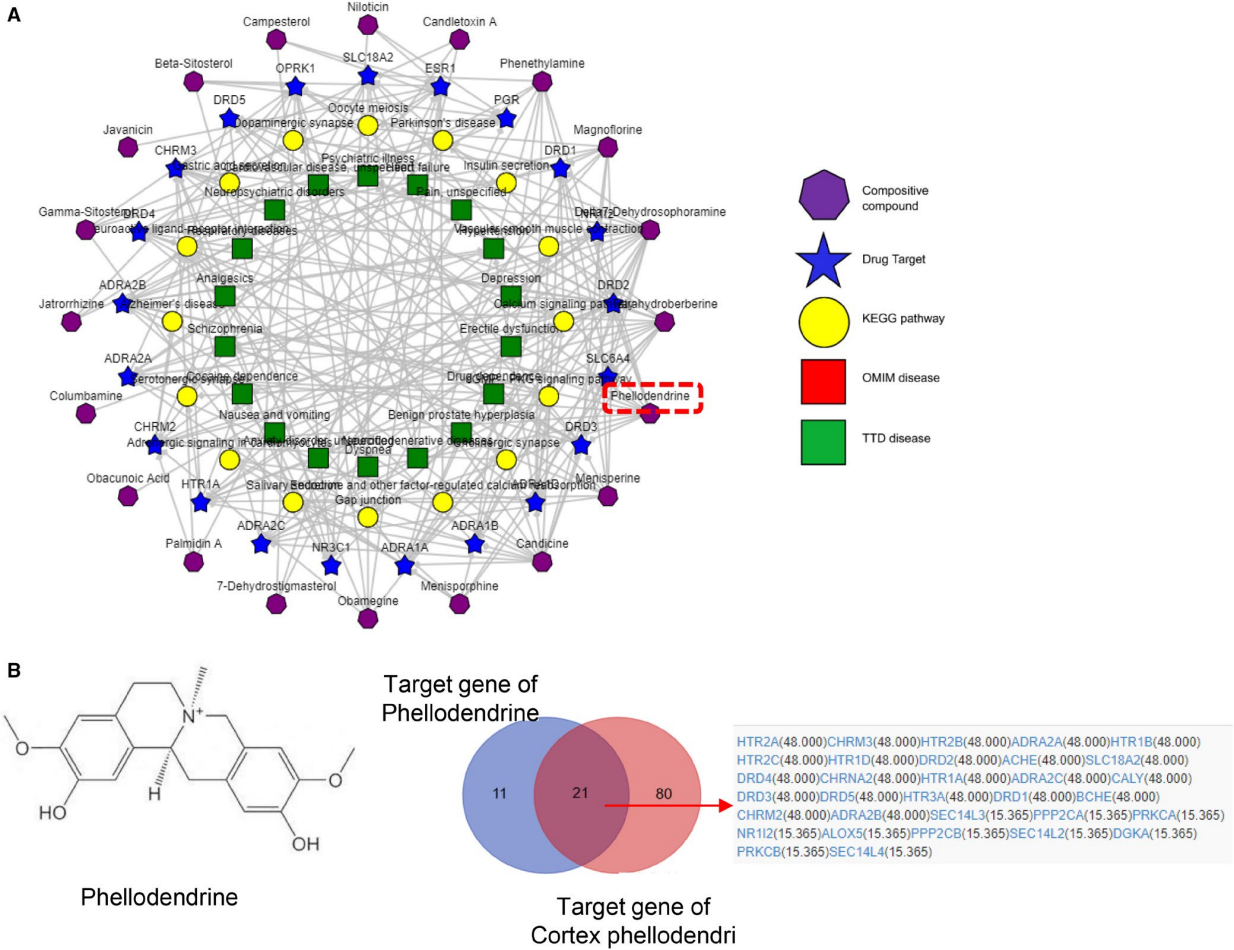
Network pharmacological analysis of Phellodendrine targets. A, Network analysis of the main components, targets and signal pathways of Cortex Phellodendri. B, Potential target of phellodendrine

The BATMAN website (http://bionet.ncpsb.org/batman‐tcm/) was consulted to reveal the main ingredients in Compound Cortex Phellodendri Liquid analyse the mechanism by which these components treat UC. The direct action protein targets in Compound Cortex Phellodendri Liquid for UC treatment were screened by constructing an interactive network. BATMAN uses a similarity‐based method to predict the potential targets of traditional Chinese medicine components, and its core idea is to sort the potential targets according to their interaction with known targets. The targets of five traditional Chinese medicines, namely forsythia, *Phellodendron*, honeysuckle, dandelion and centipede, in Compound Cortex Phellodendri Liquid are shown in Figure 2A. The KEGG pathways of these intersection targets were analysed further. KEGG enrichment analysis revealed that the targets of Compound Cortex Phellodendri Liquid mainly affect the AMPK signalling pathway and the mTOR signalling pathway (Figure 2B). The target protein interaction network was further demonstrated through Cytoscape (Figure 2C). GO analysis also revealed that the target proteins were enriched in the AMPK signalling pathway (Figure 2D). AMP‐activated protein kinase (AMPK) is a highly conserved serine/threonine protein kinase that is an energy receptor in eukaryotic cells. AMPK plays an anti‐inflammatory role by inhibiting the expression of inflammatory signalling pathways, such as NF‐κB, MAPK and JAK‐STAT, as well as inflammatory genes. AMPK is involved in the regulation of various chronic inflammatory diseases, such as inflammatory bowel disease and non‐alcoholic fatty liver disease. Therefore, the AMPK signalling pathway may be a key target of action. Phellodendrine is connected with different drug targets and signal pathways in the network pharmacologic analysis of phellodendron (shown in Figure 3A). Moreover, Figure 3B displays 21 common target genes of between phellodendrine and Compound Cortex Phellodendri Liquid, which suggested that phellodendrine is the major component of Compound Cortex Phellodendri Liquid. Determination results showing the average contents of phellodendrine were 39.08 ± 2.024 (μg/ml) determined using a validated high‐performance liquid chromatography method in Compound Cortex Phellodendri Liquid (Table S1). Thus, phellodendrine could be used as a central target of Compound Cortex Phellodendri Liquid for further research.

### 3.3 Phellodendrine therapy for UC

**FIGURE 4:**
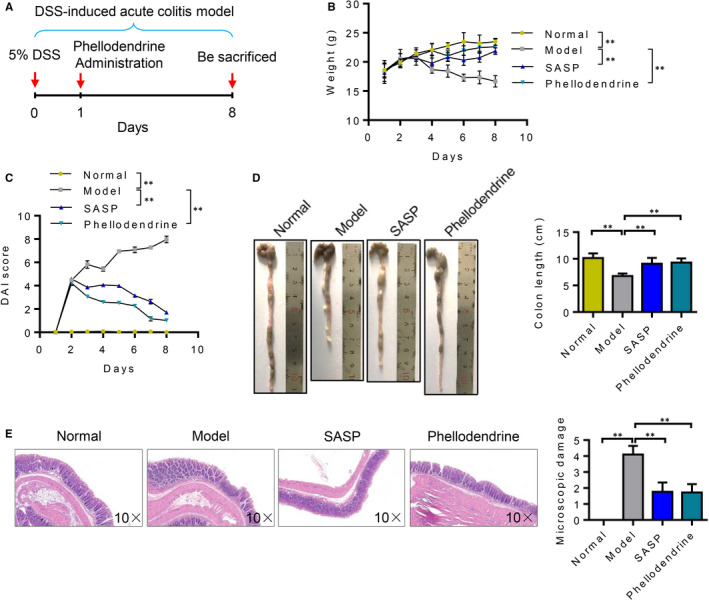
Results of animal experiments on treating ulcerative colitis with phellodendrine. A, Experimental flow charts of acute ulcerative colitis induced by DSS and treatment of mice with phellodendrine. B, Changes in the weight of mice in the control group, model group, SASP and phellodendrine treatment group. C, Changes in DAI scores of mice in the control group, model group, SASP and phellodendrine treatment group. D, Colon length and statistical analysis results of mice in the control group, model group, SASP and phellodendrine treatment group. E. HE staining results of colon tissues of mice in the control group, model group, SASP and phellodendrine treatment group. N = 5. Data are presented as means ± SD. *P < 0.05 and **P < 0.01

The efficacy of phellodendrine in treating UC was further verified. An animal model of UC was first constructed. From the 2nd day after the successful replication of the model, the mice in the normal group had lively spirits, quick reactions, bright hair, normal diet and normal urine and faeces. However, all the mice in the model group, the salazosulphapyridine group and the phellodendrine group showed mucinous stool, depression, decreased appetite, disordered hair and decreased body mass. After 3 days of treatment, two models were randomly selected from the model and the normal groups for model evaluation. The modelling was successful. As expected, compared to control group, weight loss, increasing DAI score and reduced colon length were significantly in the model group (shown in Figure 4B‐D). Besides, the disappearance of mucosal folds, mucosa inflammation and crypt lack were observed in the colon tissues of DSS‐induced model mice (Figure 4E). After administration, the body mass gradually recovered, visual blood gradually decreased, body shape gradually improved and hair and mental state gradually recuperated of the mice in both the phellodendrine and the pentaminosalicylic acid groups (Figure 4A and B). The DAI score of the model group was significantly higher than that of the normal group (*P* < 0.05). Compared with the model group, the DAI score of each treatment group significantly decreased (*P* < 0.05) (Figure 4C). Results showed that phellodendrine substantially reduced diarrhoea and blood stool in the mice induced by DSS. Colon length was reduced in the model group compared with that in the normal group. Colon length was normal in the phellodendrine treatment group (Figure 4D). Pathological results showed that the pathological damage index of the colon in the model group significantly increased (*P* < 0.05) compared with that in the normal group. However, compared with that in the model group, the pathological colonic injury index in the phellodendrine group was significantly reduced (*P* < 0.01), suggesting that phellodendrine could alleviate DSS‐induced colonic lesions in mice (Figure 4E).

### 3.4 Molecular studies on the effects of phellodendrine on autophagy

**FIGURE 5:**
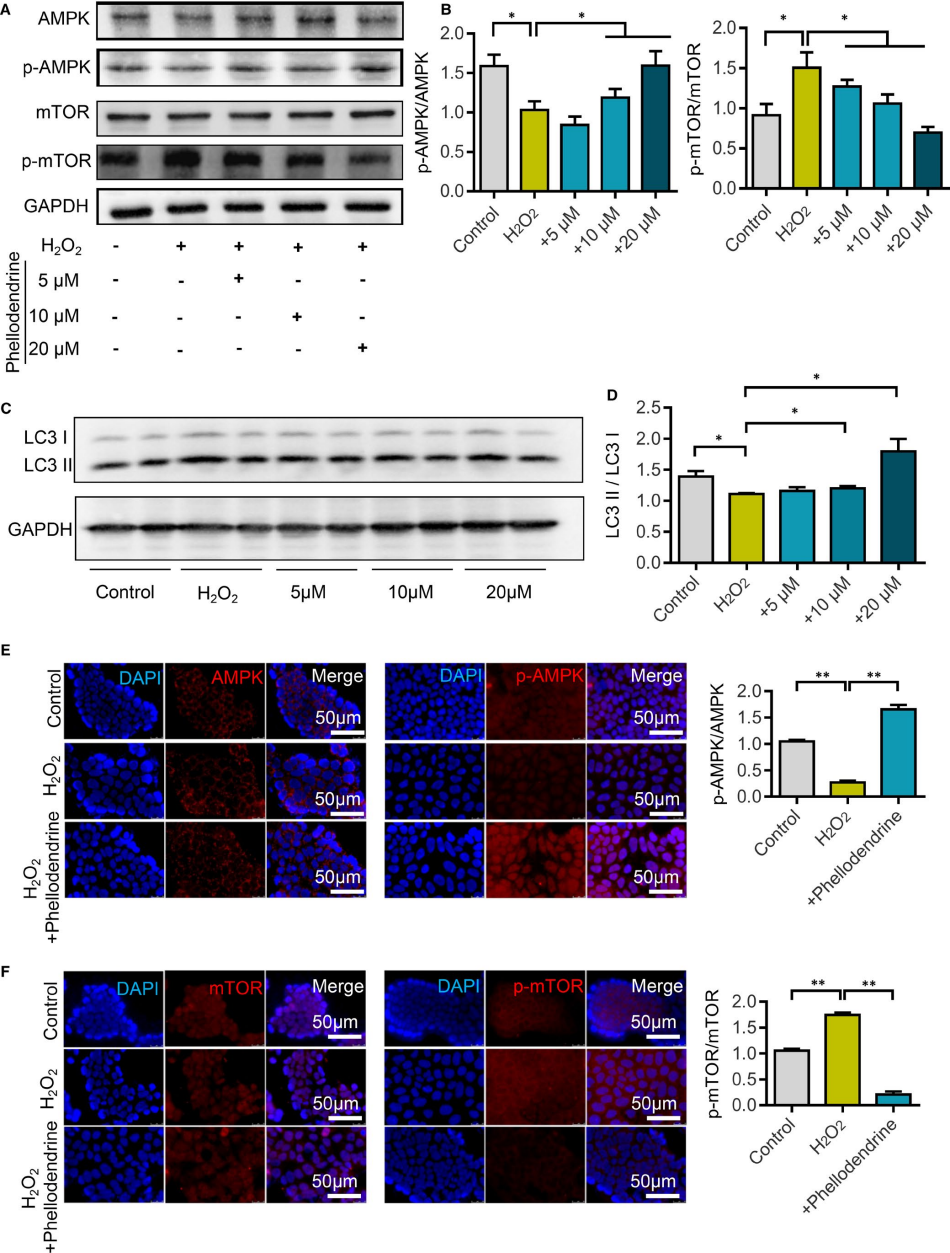
Phellodendrine promotes autophagy in ulcerative colitis cells through the AMPK/mTOR signalling pathway. A, After different treatments, the expression of AMPK, p‐AMPK, mTOR and p‐mTOR in the AMPK/mTOR signalling pathway changes. B, Statistical results of the changes in the expressions of AMPK, p‐AMPK, mTOR and p‐mTOR after different treatments. C, After different treatments, the expression levels of autophagy markers LC3I and IL3II were detected. D, Statistical results of the changes in the ratio of IL3II/LC3I after different treatments. E, Immunofluorescence staining detects changes in AMPK and p‐AMPK expression levels after different treatments. F, Immunofluorescence staining detects changes in mTOR and p‐mTOR expression after different treatments. N = 3. Data are presented as means ± SD. *P < 0.05 and **P < 0.01

First, changes in AMPK and mTOR in autophagy during intestinal cell injury were detected via Western blot. Experimental results showed that, compared with that in the control group, the ratio of p‐AMPK/AMPK decreased after H_2_O_2_ stimulation, whereas the ratio of p‐mTOR /mTOR increased, indicating that H_2_O_2_ stimulation inhibited the AMPK/mTOR signalling pathway. However, after phellodendrine treatment, the p‐AMPK/AMPK ratio increased, whereas the p‐mTOR/mTOR ratio decreased (Figure 5A and B). LCII/LCI results of autophagy markers showed that the LCII/LCI ratio decreased after H_2_O_2_ stimulation. However, the LCII/LCI ratio increased after phellodendrine treatment (Figure 5C and D), suggesting that phellodendrine treatment could promote increased autophagy. Immunofluorescence detection of autophagy in the colon cells of the mice in all groups showed that the p‐AMPK/AMPK ratio increased, whereas the p‐mTOR /mTOR ratio decreased (Figure 5E and F). Immunofluorescence results were consistent with those of Western blot.

### 3.5 Bioinformatics analysis of phellodendrine for the treatment of UC

**FIGURE 6:**
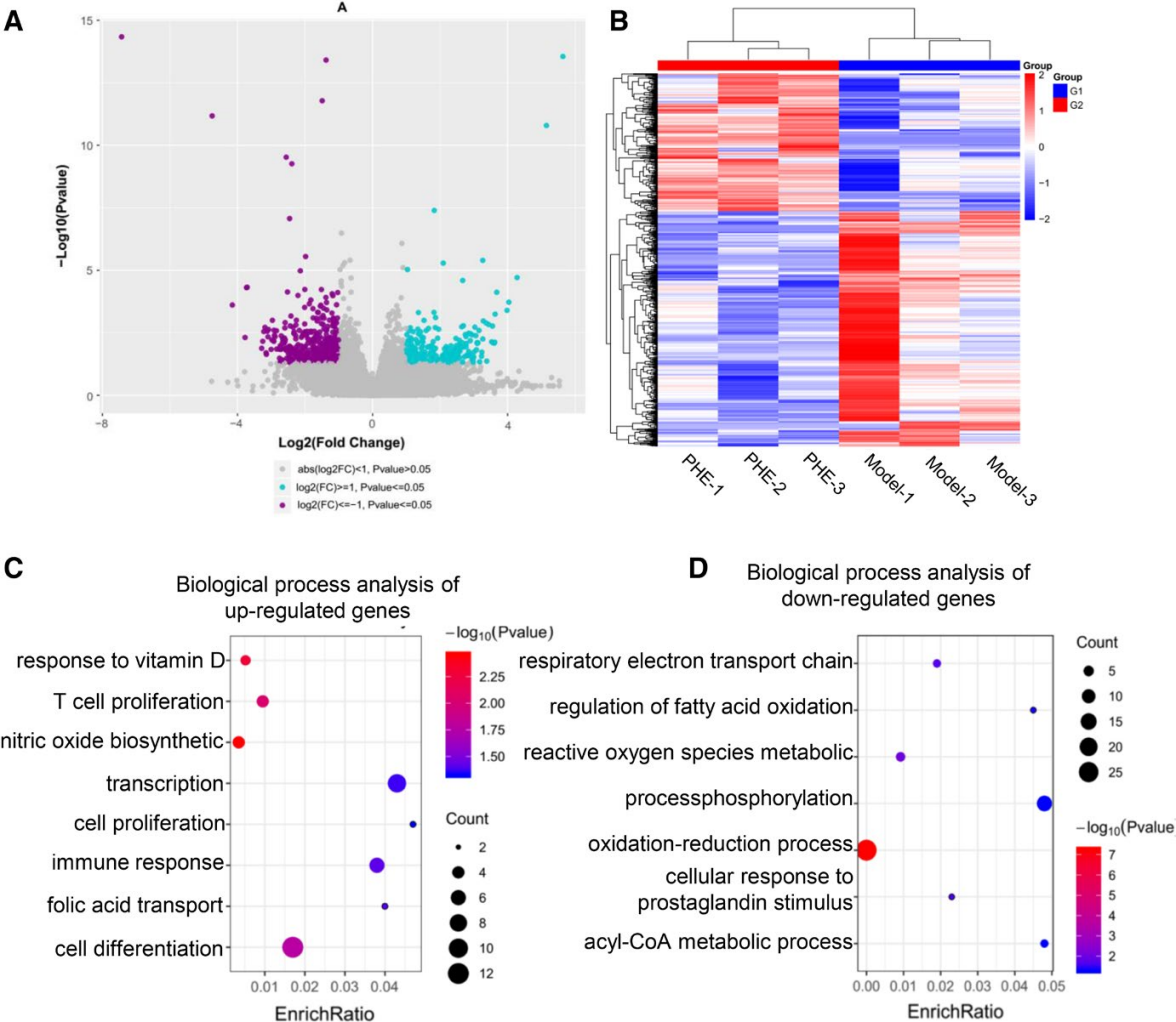
Efficacy of phellodendrine in the treatment of ulcerative colitis as determined by microarray sequencing analysis. A, Volcanograph analysis of differentially expressed genes between the control group and the phellodendrine treatment group. B, Cluster analysis of differentially expressed genes between the control group and the phellodendrine treatment group. C, Biological process analysis of up‐regulated genes. D, Biological process analysis of down‐regulated genes. Data are presented as means ± SD. *P < 0.05 and **P < 0.01

Differences in the gene expression profiles between the animal model group of UC and the phellodendrine treatment group were analysed using expression spectrum microarray. The mechanism related to the treatment of UC was further analysed by GO enrichment and KEGG analyses. The differential expression of genes in the model group and the phellodendrine treatment group was analysed using volcanic atlas. Volcanograms show up‐regulated genes (blue dots) and down‐regulated genes (purple dots). A total of 372 differentially expressed genes were found, including 131 up‐regulated genes and 241 down‐regulated genes (Figure 6A). Cluster analysis distinguished well the phellodendrine group from the control group, and the selected DEGs could be used for subsequent analysis (Figure 6B). GO enrichment analysis showed that the main metabolic processes (BP) of the up‐regulated genes were positive regulation of nitric oxide biosynthetic process, response to vitamin D, positive regulation of T cell proliferation, cell differentiation, immune response, folic acid transport, positive regulation of transcription and positive regulation of cell proliferation (Figure 6C). The primary biological processes of the down‐regulated genes were oxidation‐reduction process, reactive oxygen species metabolic process, respiratory electron transport chain, cellular response to prostaglandin stimulus, regulation of fatty acid oxidation, phosphorylation and acyl‐CoA metabolic process (Figure 6D).

### 3.6 Sequencing data of intestinal flora after phellodendrine treatment

**FIGURE 7:**
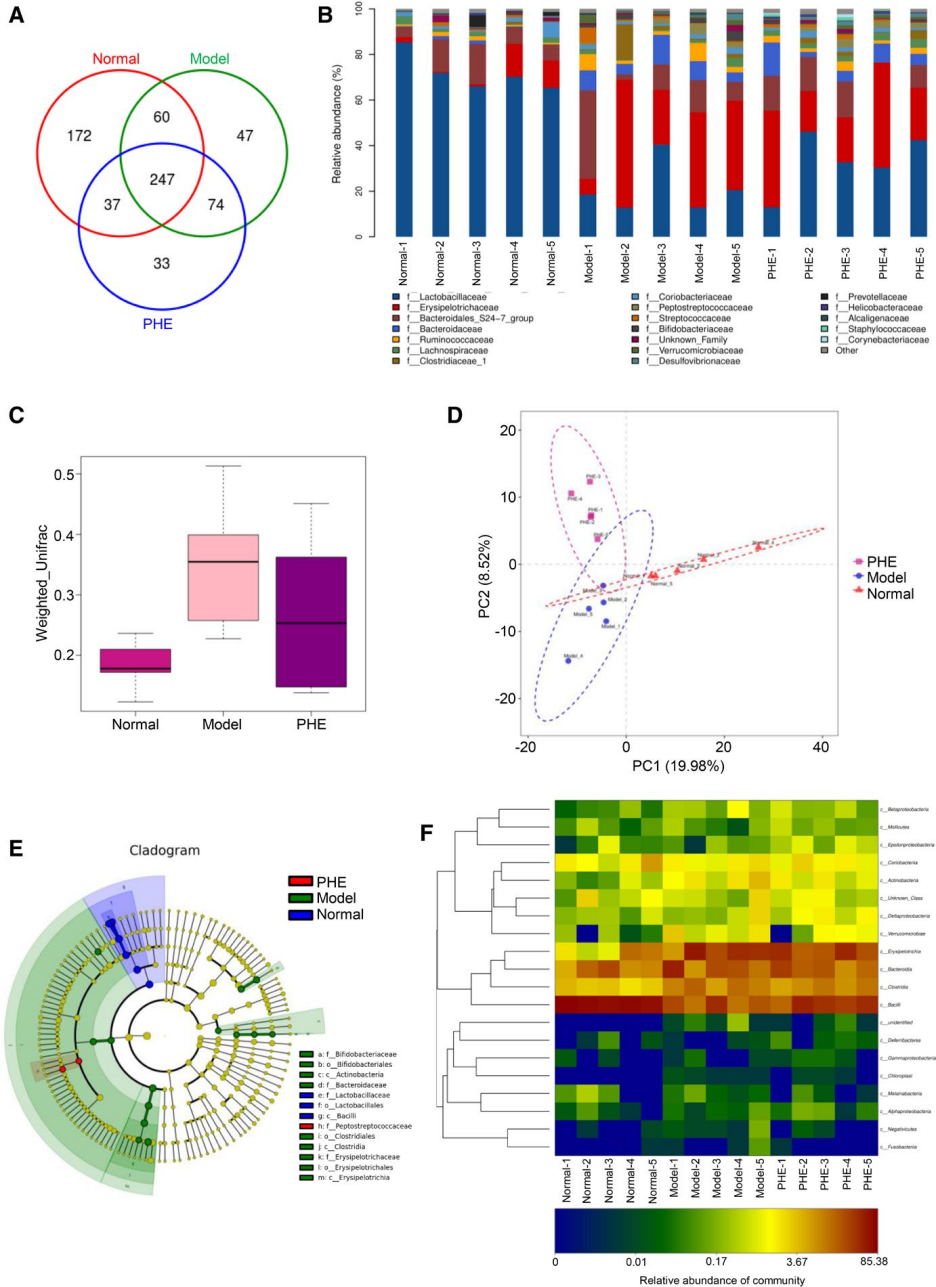
Sequencing analysis of intestinal flora of the mice with ulcerative colitis treated with phellodendrine. A, Venn diagram analysis of mouse faeces in the control group, model group and phellodendrine treatment group. Different colours represent different samples, and the area where circles of different colours overlap is an intersection, that is the same OTU that overlaps several colour circles. B, Analysis of the abundance of intestinal flora of mice in the control group, model group and phellodendrine treatment group. The abscissa is the sample name, and the ordinate is the relative abundance of the species in the sample. C, Weighted UniFrac analysis of the intestinal flora of mice in the control group, model group and phellodendrine treatment group. D, Principal component analysis of intestinal flora of mice in the control group, model group and phellodendrine treatment group. E, Evolutionary clade graph analysis based on LEfSe analysis of classification information. F, OTU and taxonomic heatmap analysis of each group of intestinal flora. N = 5

The petal plot represents the number of OTUs that are endemic and common between samples/groups. Each petal represents a sample/group, and the core number in the middle represents the number of OTUs shared by all samples. The number on the petals represents the number of OTUs unique to the sample/group (Figure 7A). The core microbiome can be found on the basis of the common OTUs of the sample and species that the OTUs represent. In Figure 7B, the abscissa is the sample name, and the ordinate is the relative abundance of species in the sample. The figure shows species with a relative abundance of over 1%. Results showed that the abundance of intestinal flora of the phellodendrine‐treated mice was more similar to that of the normal group. The dominant species were screened according to the results of species classification. Species abundance was counted by taxonomic tree, and differences in abundance and evolutionary relationships of the dominant species in each group were evaluated from the whole taxonomic system. Compared with the normal group, the abundance of Lactobacillaceae in the model group decreased. The flora of phellodendrine‐treated mice was closer to that of the normal group (Figure 7C). Partial least square discriminant analysis (PLS‐DA) is a multivariate statistical analysis method for discriminant analysis. In this study, PLS‐DA based on the OTUs showed that PCA could well distinguish the three groups of features (Figure 7D). Evolutionary branch diagrams of LEfSe analysis based on classification information are given in Figure 7E. The circle of radiation from inside to outside represents the taxonomic rank from phylum to genus (or species). Each small circle at a different classification level represents a classification at that level, and the diameter of the small circle is proportional to relative abundance. Among them, the red node represents the microbial group that plays an important role in the red group. Green nodes represent groups of microorganisms that play an important role in the green group. Other circles have similar meanings. Heatmap can reflect data information in two‐dimensional matrix or table with colour changes, and it can intuitively represent the size of data value with defined colour depth. Thermal map analysis showed that the flora of phellodendrine‐treated mice was closer to that of the normal group (Figure 7F).

## 4 DISCUSSION

Ulcerative colitis is a major type of inflammatory bowel disease, which entails diffused inflammation of colorectal mucosa.^27^ To date, this disease has no clear aetiology and a corresponding effective medical treatment.^28^ Inflammation caused by oxidation reaction is the most important factor for the onset and deterioration of UC.^29^, ^30^ Autophagy also frequently occurs in colorectal inflammation.^31^ Autophagy plays an important role in the regulation of inflammatory response.^32^ Autophagy influences the remission of infectious diseases and the pathological processes of inflammatory diseases, as well as plays an anti‐inflammatory effect in inflammatory responses induced by tissue damage without bacterial infection.^33^ Intestinal epithelial autophagy is an important mechanism for mammalian intestinal defence against invading bacteria.^34^ Blocking the autophagy pathway of epithelial cells and dendritic cells leads to an inability of the intestinal mucosal barrier to resist and clear various pathogens, such as *Salmonella typhimurium*, *Shigella* *fusarium* and invasive *Escherichia* *coli*.^35^ Autophagy inducers improve TNF‐α‐induced intestinal barrier damage by activating autophagy. Abnormalities in autophagy‐related genes can impair the intestinal mucosal barrier. These studies indicated that abnormal autophagy may be involved in the occurrence and development of UC by affecting the repair of the intestinal mucosal barrier.

LC3, which exists in the form of LC3‐I and LC3‐II2, is the most important protein molecule in autophagy and primarily involved in the formation of autophagosomes.^36^, ^37^ Detection of LC3 can reflect the integrity of autophagy flow.^38^ In this study, Western blotting revealed that phellodendrine treatment substantially promoted the increase in LC3‐II/LC3‐I compared with the model group. Therefore, phellodendrine preconditioning may play a protective role to intestinal epithelial cells by promoting autophagy.

Traditional Chinese medicine formulations play an important role in the key biological processes of the occurrence and development of diseases by acting on multiple targets through multiple components.^39^, ^40^ Owing to the rapid development of bioinformatics, network pharmacology has become a new method for the efficient and systematic determination of the molecular mechanisms of traditional Chinese medicine prescriptions.^41^ Network pharmacology is performed to determine the relationship between drugs, targets and diseases, as well as presents drug‐target networks through systematic thinking. Interaction relationships can be visualized as a network model, and the effects of drugs on biological networks can be studied from an overall perspective. In this study, the active components of Compound Cortex Phellodendri Liquid were screened via the network pharmacologic analysis method. The targets of the active compounds were further predicted, and their correlation with UC was analysed. Subsequently, a PPI network of the active ingredients and targets was visualized. The mechanism by which Compound Cortex Phellodendri Liquid treats UC was further analysed by identifying the candidate targets of the active compounds of Compound Cortex Phellodendri Liquid. The pharmacological mechanism of phellodendrine was also investigated.

Oxidative stress is a state of stress caused by the disruption of homeostasis between oxidative and antioxidant systems.^42^ Numerous studies reported that the reactive oxygen species produced in oxidative stress can induce autophagy production.^43^ Autophagy can alleviate the damage caused by oxidative stress, thus protecting cells from survival.^44^ Thus far, the mechanism by which autophagy inhibits inflammasomes remains unclear. The present study found that autophagy decreased after UC occurred. If autophagy is insufficient, then normal cell homeostasis cannot be maintained and inflammation can occur. This process indicates that autophagy plays an important role in the body's resistance to UC. This study proved that autophagy is a positive feedback that regulates UC pathogenesis, an outcome that is beneficial to the reduction of inflammation in the body. After phellodendrine intervention, the level of autophagy considerably increased, whereas cell damage substantially decreased. Therefore, the mechanism by which phellodendrine lowers oxidative stress in the colonic mucosa of UC may be related to its enhancement of autophagy (Figure S1). Using an animal model of UC, the efficacy of phellodendrine was evaluated and its potential pharmacological mechanism was investigated to provide new therapeutic strategies for UC treatment and provide a scientific basis for the clinical applications of phellodendrine and its corresponding formulations.

## 5 CONCLUSION

This study investigated the effects and molecular mechanism of phellodendrine in Compound Phellodendrine solution for UC treatment. Phellodendrine processing may promote autophagy by activating the AMPK‐mTOR signalling pathway and ultimately exert a protective effect on UC. However, whether phellodendrine regulates autophagy through other signalling pathways remains to be explored. Clarifying its specific mechanism of action in UC will provide a theoretical basis for the clinical applications of phellodendrine and new ideas for clinical treatment of UC.

## CONFLICT OF INTEREST

The authors declare no conflicts of interest.

## AUTHOR CONTRIBUTIONS

**Shuai Su:** Data curation (equal); Writing‐original draft (equal). **Xin Wang:** Data curation (equal); Writing‐original draft (equal). **Xiaonan Xi:** Data curation (equal); Writing‐original draft (equal). **Lanping Zhu:** Data curation (equal). **Qiuyu Chen:** Data curation (equal). **Hongxia Zhang:** Data curation (equal). **Yuan Qin:** Data curation (equal). **Boli Yang:** Data curation (equal). **Na** **Che:** Data curation (equal). **Hailong Cao:** Project administration (equal). **Weilong Zhong:** Data curation (equal); Project administration (equal); Writing‐original draft (equal). **Bangmao Wang:** Project administration (lead).

## Supporting information

Supinfo

## Data Availability

The data that support the findings of this study are available on request from the corresponding author. The data are not publicly available due to privacy or ethical restrictions.
