# Supplementary material for: Phellodendrine promotes autophagy by regulating the AMPK/mTOR pathway and treats ulcerative colitis
Source: J Cell Mol Med. 2021 May 18;25(12):5707–20. doi: 10.1111/jcmm.16587 (PMC8184668; doi:10.1111/jcmm.16587)
Supplement: Supplementary file 1 — Supinfo [file JCMM-25-5707-s001.doc]

Supplementary Figure 1

**
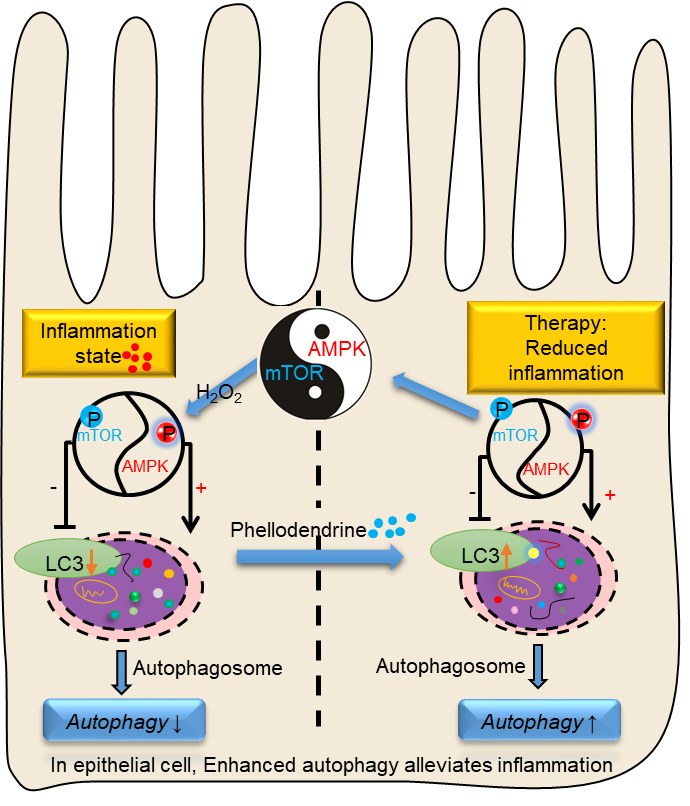
**

Phellodendrine promotes autophagy through the AMPK/mTOR signaling pathway for the treatment of ulcerative colitis.

Table S1. Determination Results of Phellodendrine in ten batches of Compound Cortex Phellodendri Liquid

| LOT No. | content (μg/ml) | LOT No. | content (μg/ml) |
| --- | --- | --- | --- |
| 16071211 | 37.7 | 17033112 | 41.4 |
| 1703041 | 35.8 | 17040112 | 42.4 |
| 17031012 | 48.4 | 17040612 | 34.2 |
| 17031312 | 47.7 | 18060711 | 30.8 |
| 17032312 | 42.1 | 18072112 | 30.3 |
